# Genomic and phenotypic landscapes of X-linked hereditary hearing loss in the Chinese population

**DOI:** 10.1186/s13023-024-03338-z

**Published:** 2024-09-13

**Authors:** Haifeng Feng, Shasha Huang, Ying Ma, Jinyuan Yang, Yijin Chen, Guojian Wang, Mingyu Han, Dongyang Kang, Xin Zhang, Pu Dai, Yongyi Yuan

**Affiliations:** 1 Senior Department of Otolaryngology Head and Neck Surgery, the 6th Medical Center of Chinese PLA General Hospital, Chinese PLA Medical School,, Beijing, 100853 China; 2 State Key Laboratory of Hearing and Balance Science, Beijing, 100853 China; 3 National Clinical Research Center for Otolaryngologic Diseases, Beijing, 100853 China; 4 Key Laboratory of Hearing Science, Ministry of Education, Beijing, 100853 China; 5 Beijing Key Laboratory of Hearing Impairment Prevention and Treatment, Beijing, 100853 China; 6 Chinese PLA Medical School, Beijing, 100853 China

**Keywords:** X-linked hearing loss, DFNX, Genetic variants spectrum, Genotype–phenotype correlations

## Abstract

**Background:**

Hearing loss (HL) is the most common sensory birth deficit worldwide, with causative variants in more than 150 genes. However, the etiological contribution and clinical manifestations of X-linked inheritance in HL remain unclear within the Chinese HL population. In this study, we focused on X-linked hereditary HL and aimed to assess its contribution to hereditary HL and identify the genotype–phenotype relationship.

**Methods:**

We performed a molecular epidemiological investigation of X-linked hereditary HL based on next-generation sequencing and third-generation sequencing in 3646 unrelated patients with HL. We also discussed the clinical features associated with X-linked non-syndromic HL-related genes based on a review of the literature.

**Results:**

We obtained a diagnostic rate of 52.72% (1922/3646) among our patients; the aggregate contribution of HL caused by genes on the X chromosome in this cohort was ~ 1.14% (22/1922), and *POU3F4* variants caused ~ 59% (13/22) of these cases. We found that X-linked HL was congenital or began during childhood in all cases, with representative audiological profiles or typical cochlear malformations in certain genes. Genotypic and phenotypic analyses showed that causative variants in *PRPS1* and *AIFM1* were mainly of the missense type, suggesting that phenotypic variability was correlated with the different effects that the replaced residues exert on structure and function. Variations in *SMPX* causing truncation of the protein product were associated with DFNX4, which resulted in typical audiological profiles before and after the age of 10 years, whereas nontruncated proteins typically led to distal myopathy. No phenotypic differences were identified in patients carrying *POU3F4* or *COL4A6* variants.

**Conclusions:**

Our work constitutes a preliminary evaluation of the molecular contribution of X-linked genes in heritable HL (~ 1.14%). The 15 novel variants reported here expand the mutational spectrum of these genes. Analysis of the genotype–phenotype relationship is valuable for X-linked HL precise diagnostics and genetic counseling. Elucidation of the pathogenic mechanisms and audiological profiles of HL can also guide choices regarding treatment modalities.

**Supplementary Information:**

The online version contains supplementary material available at 10.1186/s13023-024-03338-z.

## Introduction

Hearing loss (HL) is the most frequent sensory birth deficit worldwide: one in every 500 newborns exhibits bilateral HL [1]. Although its etiology is diverse, HL has a genetic origin in a large proportion of cases (50–60%) [1]. Heritable HL exhibits substantial heterogeneity with respect to clinical manifestations and disease-causing genes. Based on the clinical symptoms, hereditary HL can be subdivided into non-syndromic HL (NSHL), in which HL is the only symptom observed, and syndromic HL (SHL) that involves other organ or system abnormalities. In implicated genes, mutations in more than 150 genes have been identified to cause hereditary HL (Hereditary Hearing Loss Homepage: http://hereditaryhearingloss.org). Among these genes, the X-linked genes are considerably rarer than autosomal genes; only six known X-linked NSHL (DFNX) loci and five genes have been identified: *PRPS1* (DFNX1, MIM#304500) [2], *POU3F4* (DFNX2, MIM #304400) [3], *SMPX* (DFNX4, MIM#300066) [4, 5], *AIFM1* (DFNX5, MIM#300614) [6], and *COL4A6* (DFNX6, MIM#303630) [7]. Many syndromic X-linked disorders are associated with HL, such as Norrie syndrome and Alport syndrome involving HL as a major clinical finding [8]. However, the etiological contributions and audiological phenotypes of X-linked hereditary HL have rarely been studied, especially in the Chinese HL population.

Locus heterogeneity results in complexity regarding DNA diagnostics of a Mendelian disorder, such as hereditary forms of HL. The molecular contributions of certain genes in the deaf population remain unclear, especially with respect to genes associated with X-linked HL that have not been systematically investigated. Furthermore, due to the low mutation frequencies of X-linked genes and the lack of clear genotype–phenotype correlations, it is extremely time-consuming and expensive to use conventional gene-by-gene Sanger sequencing for variant identification in these genes. Therefore, the identification of etiological contributions responsible for heritable X-linked HL has been a major challenge for geneticists and otologists. This limitation, however, may be overcome by the emergence of next-generation sequencing (NGS) technologies [9]. NGS complements the disadvantages of traditional sequencing method; it has enabled detection of nearly all causative genes and variants involved in the onset and development of HL, including the less commonly screened X-linked genes, in a high-throughput and cost-effective manner. In addition, advances in third-generation sequencing (TGS) have enhanced the molecular diagnosis of inherited HL in some cases.

In the present study, we focused on X-linked inheritance of HL to assess its aggregate contribution to hereditary HL by NGS and TGS in a large cohort of HL patients from China. In addition, we reviewed the literature regarding clinical phenotypes resulting from variants in DFNX genes to investigate genotypic and phenotypic features. These results enhance the overall understanding of the molecular etiology of HL in the Chinese population and provide a systematic evaluation of the etiological contribution and audiological profiles of X-linked HL. Identification of the genetic basis of X-linked HL is also essential for providing proper genetic counseling to patients and their families, and it will influence diagnostics, preventive, and treatment decisions.

## Materials and methods

### Study participants and clinical evaluations

Probands with HL were recruited into this study from among consecutive patients referred to the otolaryngology department for clinical genetic testing and counseling during the period from 2015 to 2023. Clinical information was collected from probands or their legal guardians through a questionnaire regarding the following aspects of their condition: family history, age at onset of HL, progression, noise or aminoglycoside exposure, and other relevant clinical manifestations. Some cases involving secondary HL, such as trauma and otitis media, were also excluded. Multidisciplinary examinations were conducted to identify patients with SHL. High-resolution computed tomography (HRCT) or magnetic resonance imaging of the temporal bones was performed as necessary to evaluate middle or inner ear malformations. Pure tone audiometry (PTA) was measured to define the HL type and severity. For individuals who could not undergo PTA, an objective audiometry examination was conducted, such as auditory brainstem response (ABR). Other audiometric testing techniques, such as distortion product otoacoustic emission (DPOAE), were performed as necessary. The severity of HL was evaluated by averaging the thresholds of PTA at 0.5, 1, 2, and 4 kHz, or the ABR response threshold in the ear with better hearing. Severity was subdivided as follows: mild HL, 26–40 dB; moderate HL, 41–70 dB; severe HL, 71–90 dB; and profound HL, > 90 dB [10].

The study protocol was approved by the Ethics Review Committee of Chinese PLA General Hospital (Approval number S2018-088–01), and all procedures involving human participants complied with the tenets of the Declaration of Helsinki. Prior to enrollment in the study, all included individuals or their legal guardians provided written informed consent to participate.

### Molecular genetics

Whole blood (2–3 mL) was collected from all probands. Genomic DNA was extracted from peripheral blood leukocytes using a GenMagBio Genomic DNA Purification kit (GenMagBio, Changzhou, China), in accordance with the manufacturer’s instructions. Genetic screening was performed by whole-exome sequencing (WES) or using a predefined panel of 227 human deafness-related genes (Additional file 1: Table S1). DNA fragmentation, end-repair, targeted enrichment, targeted region captures, and library construction were performed in accordance with established procedures, as reported thoroughly in previous studies [11, 12]. Regions with lower coverage were subjected to additional Sanger sequencing. Trimming of sequencing adapters and inferior reads with Trimmomatic, and mapping of cleaned reads to the human reference genome (version GRCh37) using the Burrow-Wheeler Aligner (version 0.7.17-r1188). Duplicate reads were flagged by Sambamba [13] (version 0.6.6). Single-base variations and small insertions or deletions were investigated with HaplotypeCaller in Genome Analysis Toolkit version 4 [14]. Bioinformatics analysis was performed in the framework of bcbio‐nextgen (https://github.com/bcbio/bcbio‐nextgen), which provides best‐practice pipelines for variant calling, annotation, and validation. Candidate variants were annotated based on snpeff and vcfanno with several databases for variant frequencies in the general population. We filtered out variants with minor allele frequency > 0.05 and at least 2,000 alleles were observed in any general continental population in gnomAD database. The American College of Medical Genetics (ACMG) standards and guidelines and ClinGen Hearing Loss Clinical Domain Working Group expert specification were employed for sequence variation interpretation and the pathogenicity of the identified variants [14, 15]. TGS was performed when NGS failed to identify pathogenic variants in a patient exhibiting a specific phenotype, such as inner ear incomplete partition type III (IP-III) caused by a *POU3F4* mutation. A DNA sample from a family member, when available, was used to confirm candidate variants, co-segregation, and de novo status by PCR and Sanger sequencing.

### Genotype and phenotype analyses

**Fig. 1:**
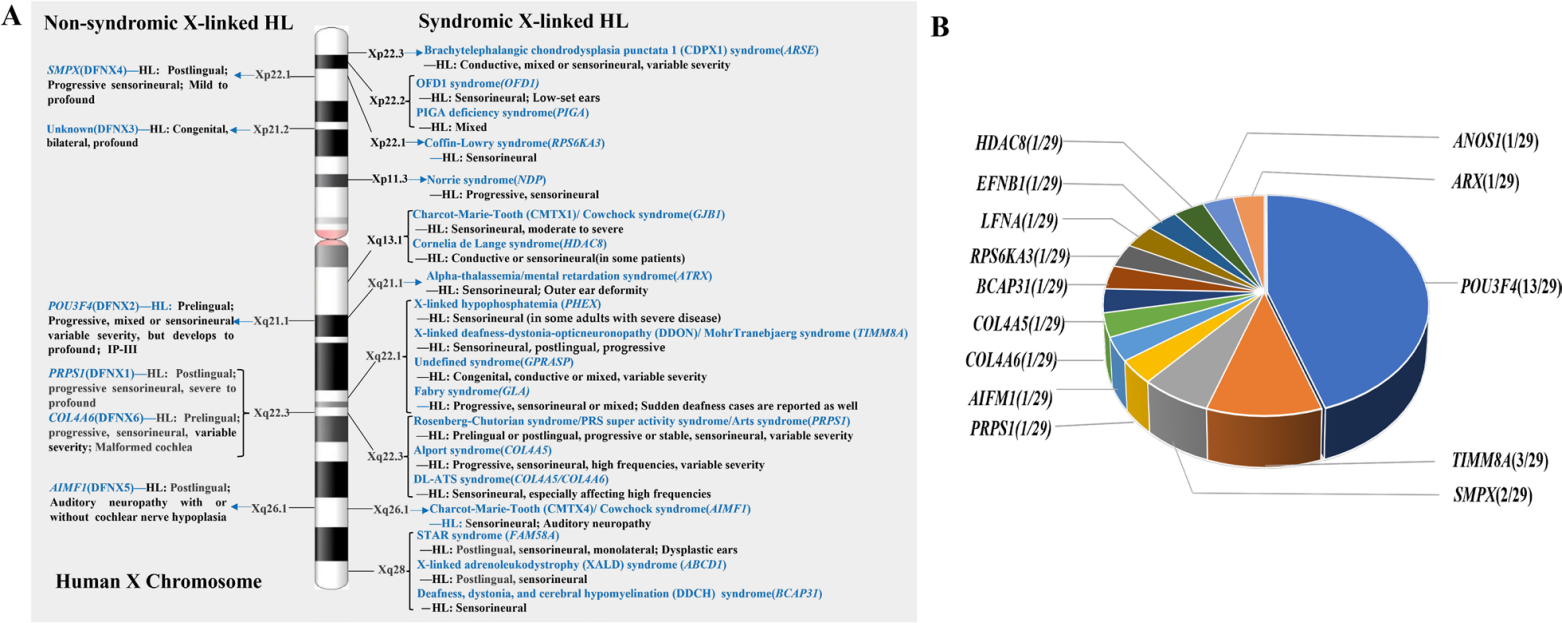
Deafness-related genes on the X chromosome. **A** Graphical representation of diseases and loci associated to syndromic (right side) and non-syndromic (left side) forms of X-linked HL. **B** X-linked genes and their proportions identified in the Chinese HL population

Studies of genes associated with syndromic and non-syndromic forms of X-linked HL published up to October 2023 were retrieved using NCBI PubMed and HGMD. The locations of these genes on the X chromosome and their corresponding diseases were summarized in Fig. 1A. The variant and disease spectra of DFNX genes were plotted, including *PRPS1* [2, 16–19], *POU3F4* [20, 21], *SMPX* [4, 5, 22–27], and *AIFM1* [7, 28–30]. Then, we screened titles, abstracts, and full texts from relevant literature to evaluate the content of the articles and extract valuable information to discuss genotype–auditory phenotype correlations. The mutation type, age at onset, and audiological profiles of HL were analyzed; sex-related differences were also examined.

## Results

### Causative genes for X-linked HL

We recruited 3646 consecutive unrelated probands with HL during the study period and performed genetic evaluation using WES (821 cases) or a predefined panel containing 227 HL-related genes (2825 cases). The data mapped to the targeted region have a mean depth of ~ 149 fold, and the coverage of 1×, 5×, 10×, and 20× was 99%, 98.7%, 98%, and 97%, respectively. The capture rate is nearly 100%. Due to NGS failure, the causative variant in *POU3F4* could not be identified in a patient exhibiting typical IP-III who underwent TGS. Of the 3646 HL patients, genetic causes were identified in 1922 (52.72%); the remaining were considered undiagnosed. The etiological factors contributing to X-linked hereditary HL in the cohort were evaluated; the identified genes and variants were recorded (Fig. 1B and Table 1).

**Table 1:** Identified variants in X-linked genes and its clinical features in Chinese HL probands

Case	Genetic alterations				ACMG		Genetic testing and clinical information			
	Gene	Transcript	Nucleotide	AA change	Evidences	LP/P/VUS	Testing	Sex/FH	Onset	Phenotypes and treatment
1	*PRPS1*	NM_002764.3	c.838A>G^#^	p.K280E	PM1 + PM2_supporting + PP1_Moderate + PP3	LP	WES	M/Yes	< 10y	Severe, SNHL; HA
2	*POU3F4*	NM_000307.5	c.232C>T	p.Q78*	PVS1 + PM2_supporting + PP4 + PP1	LP	Panel	M/Yes	Congenital	Profound, SNHL, IP-III; CI
3	*POU3F4*	NM_000307.5	c.341dup^#^	p.A116Gfs*77	PVS1 + PM2_supporting + PP4	LP	Panel	M/No	Congenital	Profound, SNHL, IP-III; CI
4	*POU3F4*	NM_000307.5	c.344_345insGGCCA^#^	p.S117Rfs*27	PVS1 + PM2_supporting + PP4 + PP1	LP	Panel	M/Yes	Congenital	Severe, SNHL, IP-III; CI
5	*POU3F4*	NM_000307.5	c.441del^#^	p.H147Qfs*94	PVS1 + PM2_supporting + PP4	LP	Panel	M/No	Congenital	Profound, SNHL, IP-III; CI
6	*POU3F4*	NM_000307.5	c.592G>C^#^	p.A198P	PM2_supporting + PP4 + PP3 + PM6 + PP1	LP	Panel	M/Yes	Congenital	Profound, SNHL, IP-III; CI
7	*POU3F4*	NM_000307.5	c.598_602del^#^	p.F201Tfs*23	PVS1 + PM2_supporting + PP4	LP	Panel	M/No	Congenital	Profound, SNHL, IP-III; CI
8	*POU3F4*	NM_000307.5	c.631A>G^#^	p.T211A	PM2_supporting + PM5 + PP3 + PP4 + PM6	LP	Panel	M/No	Congenital	Profound, SNHL, IP-III; CI
9	*POU3F4*	NM_000307.5	c.634C>T^#^	p.Q212*	PVS1 + PM2_supporting + PP4 + PP1_Strong	P	WES	M/Yes	Congenital	Profound, SNHL, IP-III; CI
10	*POU3F4*	NM_000307.5	c.856G>T^#^	p.E286*	PVS1 + PM2_supporting + PP4	LP	Panel	M/No	Congenital	Profound, SNHL, IP-III; CI
11	*POU3F4*	NM_000307.5	c.964G > A	p.V322M	PM2_supporting + PM5 + PP3 + PM6 + PP4	LP	Panel	M/No	Congenital	Severe, SNHL, IP-III; CI
12	*POU3F4*	NM_000307.5	c.968G>C^#^	p.R323P	PM2_supporting + PM5_strong + PP3 + PP4	LP	Panel	M/No	Congenital	Profound, SNHL, IP-III; CI
13	*POU3F4*	NM_000307.5	c.973del	p.W325Gfs*12	PVS1 + PM2_supporting + PP4 + PP1	LP	WES	M/Yes	Congenital	Profound, SNHL, IP-III; CI
14	*POU3F4*	NM_000307.5	~ 165 Kb Del^#^ (Chr X: 81,839,467–82,004,842)	–	PVS1 + PM2_supporting + PP4	LP	TGS	M/No	Congenital	Profound, SNHL, IP-III; CI
15	*SMPX*	NM_014332.3	c.29insA	p.N10Kfs*3	PVS1 + PM2_supporting + PP1_ Strong	P	Panel	F/Yes	Newborn	Moderate, SNHL; HA
16	*SMPX*	NM_014332.3	c.132+1G>T^#^	–	PVS1 + PM2_supporting + PM6	LP	WES	M/No	8y	Moderate, SNHL; HA
17	*AIFM1*	NM_004208.4	c.1030C>T	p.L344F	PS1 + PM2_supporting + PM1 + PP3	LP	WES	M/No	16y	Mild, AN; NA
18	*RPS6KA3*	NM_004586.3	c.328C > T	p.R110*	PVS1 + PM2_supporting + PM6	LP	WES	M/No	Congenital	Severe, SNHL, CDS; HA
19	*COL4A5*	NM_000495.5	c.3940C>T	p.Q1314*	PVS1 + PM2_supporting	LP	Panel	M/No	Congenital	Severe, SNHL, ASI; NA
20	*TIMM8A*	NM_004085.4	c.133-2A>G	–	PVS1 + PM2_supporting	LP	Panel	M/No	2.5y	Moderate, AN; HA
21	*TIMM8A*	NM_004085.4	c.238C>T	p.R80*	PVS1 + PM2_supporting + PM6	LP	WES	M/No	Congenital	Severe, SNHL; NA
22	*TIMM8A*	NM_004085.4	c.223C>T	p.Q75*	PVS1 + PM2_supporting	LP	WES	M/No	Children	Profound, SNHL; NA
23	*COL4A6*	NM_033641.4	c.1456G>A^#^	p.G486S	PM2_supporting	VUS	Panel	M/No	Congenital	Profound, SNHL, Malformed cochlea; CI
24	*EFNB1*	NM_004429.5	c.827G>A	p.R276Q	PM2_supporting + PP1	VUS	WES	M/Yes	11y	Severe, SNHL, CD; HA
25	*HDAC8*	NM_018486.3	c.512G>A^#^	p.R171H	PM2_supporting + PP3 + PM6	VUS	WES	M/No	Congenital	Profound, SNHL, CDLS; NA
26	*BCAP31*	NM_001256447.2	c.713A>T	p.D238V	PM2_supporting + PP1	VUS	WES	M/Yes	Congenital	Profound, SNHL; CI
27	*FLNA*	NM_001110556.2	c.6353C>G^#^	p.P2118R	PM2_supporting + PP3	VUS	WES	M/No	7y	Severe, SNHL; NA
28	*ARX*	NM_139058.3	c.1444G>A	p.G482S	PM2_supporting + PP3	VUS	WES	M/No	Congenital	Profound, SNHL; CI
29	*ANOS1*	NM_000216.4	c.1309C>A	p.Q437K	PM2_supporting + PM6	VUS	WES	M/No	Congenital	Profound, SNHL; CI

M, male; F, female; y, years old; P/LP, pathogenic/likely pathogenic variants; VUS, variants of uncertain significance; AA, amino acid; FH, family history; CI, cochlear implants; HA, hearing aids, IP-III, incomplete partition type III; CLS, Coffin–Lowry syndrome; ASI, Alport syndrome I; CD, craniofrontonasal dysplasia; CDLS, Cornelia de Lange syndrome

^#^These variants are not yet present in available deafness databases or the published literature

14 genes on the X chromosome were identified in the selected probands. Nearly half of all variants (13/29, 44.83%) occurred in the *POU3F4* gene, including one ~ 165-kb deletion (Chr X: 81,839,467–82,004,842) encompassing the entire *POU3F4* gene identified by TGS. Five variants were found in *PRPS1*, *SMPX*, *AIFM1*, and *COL4A6* (Table 1). The remaining 11 patients carried variants in the genes responsible for X-linked SHL, and the most common is *TIMM8A.* Based on the ACMG evidence, two mutations were attributed to pathogenic variants in two genes (*POU3F4* and *SMPX*), 20 were classified as likely pathogenic variants in seven genes (*POU3F4*, *SMPX*, *PRPS1*, *AIFM1*, *COL4A5*, *RPS6KA3*, and *TIMM8A*), and seven were variants of uncertain significance (VUS) in seven genes (*EFNB1*, *HDAC8*, *BCAP31*, *FLNA*, *COL4A6*, *ARX*, and *ANOS1*). Overall, targeted NGS and TGS identified 22 pathogenic/likely pathogenic variants in seven X-linked genes as the genetic cause of HL in 22 patients, 12 of which were novel (Table 1). The aggregate contribution of genes on the X chromosome to HL in this cohort was ~ 1.14% (22/1922); *POU3F4* variants were responsible for ~ 59% (13/22).

### Clinical findings of patients with X-linked HL

**Fig. 2:**
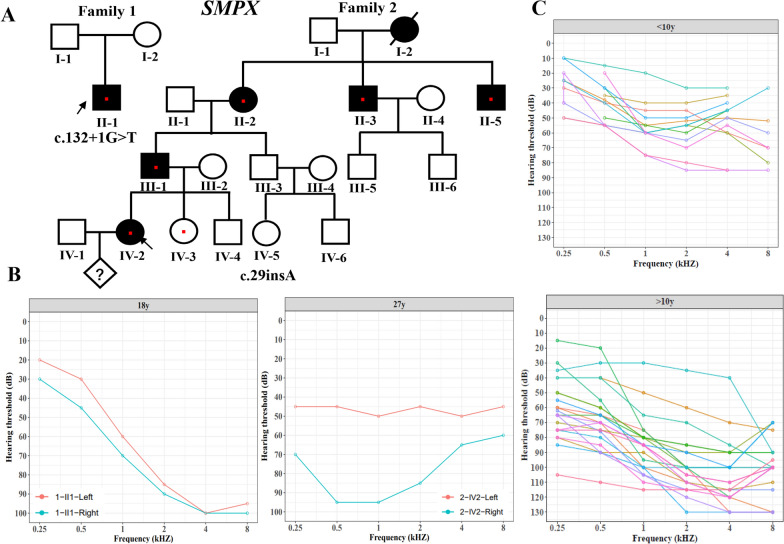
Pedigrees and audiometric characteristics of patients with *SMPX* variants. **A** Pedigrees of two deaf probands harboring *SMPX* variants; **B** audiogram of patient 1-II-1 and 2-IV-2; **C** representative audiograms (air conduction) of affected men at different ages

Twenty-nine probands with variants in X-linked genes were evaluated in the present study. The available phenotypes and family histories of the deaf probands were displayed in Table 1. The ages of the probands varied between a few months to over 34 years (mean 9.7 years, 95% confidence interval 6.0–13.5 years). However, the age at onset of HL was primarily congenital or early onset (< 6 years); only four probands presented with late-onset HL (< 18 years). Most individuals had severe to profound bilateral HL (25/29). In two families with *SMPX* variants, the affected individuals showed moderate to severe HL and presented with variability in audiometric configurations (Fig. 2A, B). Auditory neuropathy (AN) was also diagnosed in two patients by the absence or severe abnormalities of ABR and the presence of DPOAE.

Among the selected individuals, inner ear malformations were detected in 15 patients by HRCT. Thirteen patients harboring *POU3F4* variants exhibited typical IP-III mainly characterized by the absence of the cochlear modulus, presence of interscalar septa, dilatation of the internal auditory canal (IAC), and a missing bony separation between the basal turn of the cochlea and the IAC. A patient with a c.3940C > T variant in *COL4A5* had a common cavity malformation defined as a single, ovoid, or round chamber, representing the cochlea and vestibule. The remaining patient, with a c.1456G > A variant in *COL4A6*, exhibited cochlear hypoplasia and profound sensorineural HL. Under multidisciplinary examination, four patients showed syndromic features, including short stature, mental retardation, growth retardation, and cleft palate; they were diagnosed with Coffin–Lowry syndrome (CLS), Alport syndrome I (ASI), Craniofrontonasal dysplasia (CD), and Cornelia de Lange syndrome (CDLS), respectively (Table 1). The remaining patients harboring variants in genes responsible for SHL displayed no other phenotype and were diagnosed with NSHL mimic. With comprehensive evaluation, suitable auditory rehabilitation modalities were also recommended. Hearing aids (HA) are beneficial to some patients. For most affected individuals, cochlear implants (CI) were selected when HA did not substantially improve hearing (Table 1). Their hearing abilities were improved after CI surgery, which facilitated the fulfillment of learning and communication needs.

### Genotype and phenotype analysis in DFNX genes

**Fig. 3:**
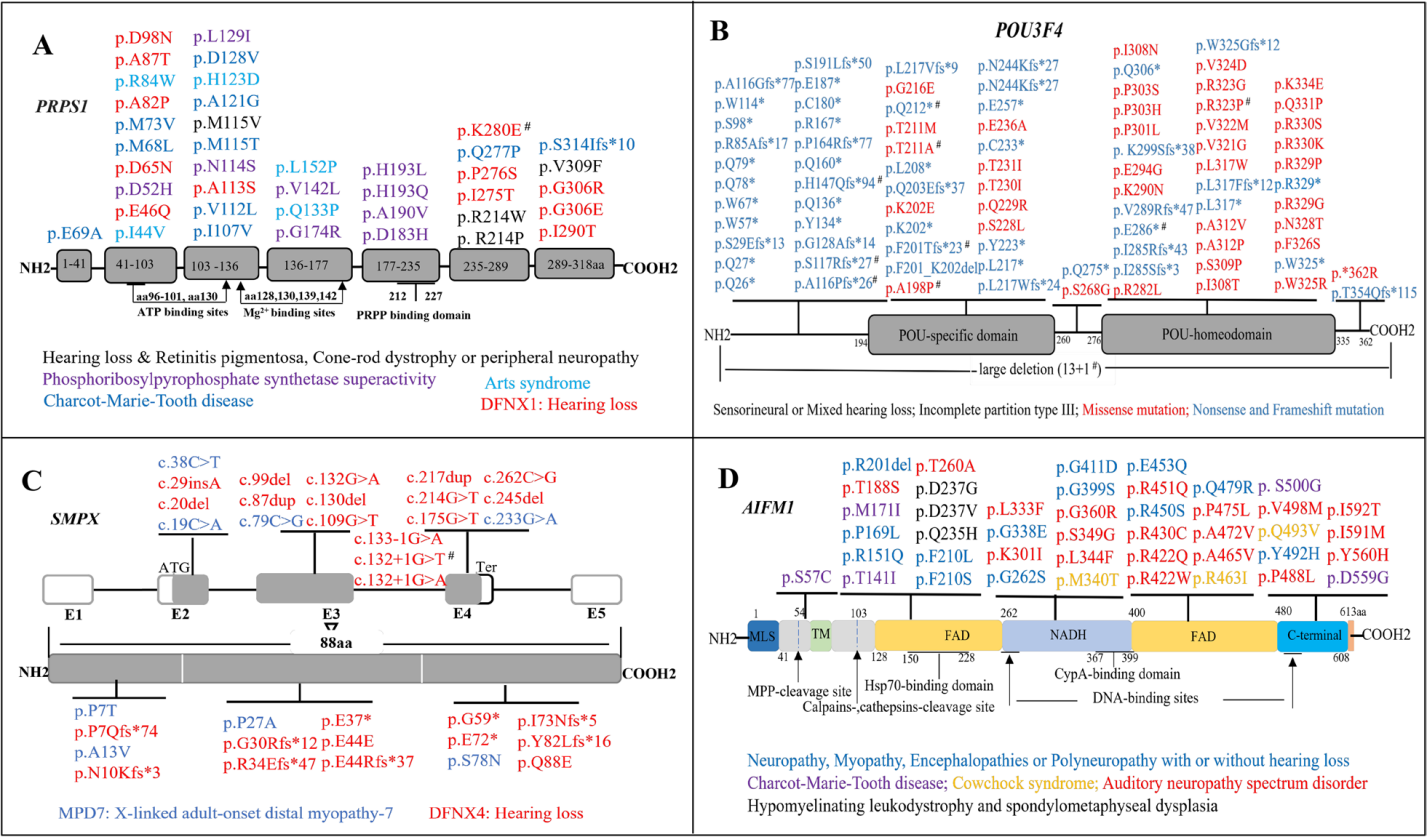
The variant and disease spectra of DFNX genes. **A** *PRPS1* variants and associated diseases; **B** the variant spectrum of *POU3F4* gene; **C** *SMPX* variants cause X-linked NSHL or adult-onset distal myopathy. **D** *AIFM1* variations in auditory neuropathy as well as syndromes. #: The variants were reported in this study

NSHL caused by changes in genes located on the X chromosome is very rare (Fig. 1A). Audiological phenotypes in these genes are diverse; pathogenic changes in certain genes can cause typical clinical characteristics. In addition, X-linked NSHL exhibits also considerable sex-related phenotypic differences due to skewed X chromosome inactivation. Figure 3 shows the clinical disorders and variant spectra of DFNX genes. The main characteristics and genes identified for each form are outlined below in detail.

### *PRPS1 *(DFNX1)

Defects in *PRPS1* can cause a wide range of disorders, from isolated HL to severe congenital syndrome (Fig. 3A). 30 mutations in the *PRPS1* coding region have been reported thus far: 11 cause DFNX1, 10 cause Charcot-Marie-Tooth disease-5 (CMTX5), 9 cause Phosphoribosyl pyrophosphate synthetase 1 (PRS-1) superactivity, 5 cause Arts syndrome, and 4 cause other phenotypes (e.g., retinitis pigmentosa, cone-rod dystrophy, or peripheral neuropathy with HL). With the exception of c.937_940dup, all of these are missense mutations, indicating that this phenotypic variety is correlated with distinct effects involving the replacement of a residue(s) in the structure of PRS-1. With regard to audiological phenotypes, the age at onset in males is variable: congenital or the first to second decade of life. Male patients with DFNX1-related mutations exhibited progressive HL, ranging in severity from moderate to profound, with highly diverse audiogram findings. Obligate female carriers had either symmetric or asymmetric HL that began in the fourth to fifth decade and varied in severity from mild to moderate. Normal hearing or unilateral HL was also observed in female individuals (Additional file 2: Table S2).

### *POU3F4 *(DFNX2)

Deafness segregating at the DFNX2 locus is associated with mutations in the *POU3F4* gene. Nearly 100 mutations, including copy number variants, have been associated with DFNX2 (Fig. 3B). These mutations are invariably located in the POU-specific domain and homeodomains of *POU3F4.* However, mutations in the POU-specific domain and its upstream region mainly cause protein truncation (37/47), whereas mutations in POU homeodomains mostly resulted in nontruncated protein (26/37). Clinical features resulting from *POU3F4* mutations include HL and typical cochlear malformation. Affected males often exhibit congenital, mixed or sensorineural, severe to profound HL. Temporal bone CT images indicated typical IP-III characterized by a thickened stapes footplate, hypoplasia of the cochlear base, absence of the bony modiolus and internal acoustic meatus, as well as abnormally wide communication between the internal acoustic meatus and the basal turn of the cochlea. However, no similar symptoms were observed in female carriers; less severe HL alone was observed in a few individuals [31].

### *SMPX *(DFNX4)

*SMPX* is the only gene responsible for X-linked dominant NSHL, and its mutations cause DFNX4. In total, 15 causative variants of *SMPX* have been associated with DFNX4 (Fig. 3C). Of these variants, 10 result in truncated SMPX proteins, three cause abnormal splicing, and the remaining two consist of a missense mutation and a synonymous mutation. In addition, four missense mutations presumably cause distal myopathy. With regard to audiological phenotypes, HL begins in the first to second decade of life in males and in the third to fifth decade of life in females. Affected males present with symmetric, progressive HL, which is initially found predominantly at high frequencies and later involves the low and middle frequencies. Generally, HL severity is mild to moderate before the age of 10 years; it subsequently progresses to severe to profound (Fig. 2C). In affected females, HL was less severe than in male patients, varying from normal to moderate. The audiological profiles in HL are diverse; unilateral, symmetric or asymmetric, stable or progressive HL can be observed (Additional file 3: Table S3).

### *AIFM1 *(DFNX5)

Mutations in *AIFM1* result in various disorders, including X-linked SNHL and SHL (Fig. 3D). 43 mutations have been reported in *AIFM1* thus far. Remarkably, several variations responsible for AN tend to occur at the NADH second FAD and C-terminal domain (17/19); none of the AN-related variations showed overlap with other *AIFM1* variations considered responsible for syndromes. With regard to audiological profiles, the ages at onset of patients with late-onset AN were mostly in the range of 5–20 years with similar clinical manifestations (Additional file 4: Table S4). HL began with low frequencies, then displayed ascending audiometric configurations and progressively worsened over time [6, 28]. Cochlear nerve hypoplasia (CNH) and delayed peripheral sensory neuropathy (presenting as extremity numbness, unsteadiness, and areflexia) were also detected in some individuals with AN. Intriguingly, all female patients had the same variant, c.1030C>T, and no phenotypic differences were present in male or female individuals with AN, suggesting that AIFM1-related AN has an X-linked dominant inheritance pattern [28].

### *COL4A6 *(DFNX6)

The *COL4A6* gene is responsible for X-linked NSHL leading to DFNX6; only three variants have been reported thus far, including c.1771G>A [7], c.3272 G>C [32], and c.951+1G>T [32]. No other hereditary diseases have been associated with mutations in *COL4A6.* The audiological features caused by *COL4A6* variants show considerable variability in terms of severity and onset among male and female patients. Affected male individuals tend to present with congenital, severe to profound sensorineural HL, whereas female individuals show delayed-onset, normal to mild or moderate HL. In addition, all affected males in a Hungarian family exhibited the c.1771G>A mutation segregating with cochlear malformation, but normal morphology of the temporal bone was detected in female carriers [7].

## Discussion

### Frequency and variant spectrum

The majority of cases of heritable HL are caused by variants in autosomal genes; X-linked genes are estimated to cause between 1 and 5% [33]. X-linked HL may present as part of a syndrome or in a non-syndromic form (Fig. 1A); there have been only a few reports regarding X-linked types of HL. William Wilde was the first to notice the excess of males among deaf [33]. Reanalysis of these data led Reardon to suggest that 5% of prelingual male deafness was X-linked [34]. Fraser stated that 6% of male prelingual genetic deafness and 1.7% of all prelingual deafness was X-linked [35]. Although X-linked HL was also mentioned in other studies [11, 36], including from Chinese deaf population, its aggregate contribution to hereditary HL, variant spectrum, and phenotype-genotype relationship were rarely evaluated in detail. In the present study, we explored the etiological contribution and variant spectrum of X-linked HL based on a substantial cohort of 3646 unrelated Chinese patients. Consistent with our assumptions of heterogeneity and low mutation frequencies of the less commonly screened deafness-related genes, NGS and TGS identified only 22 pathogenic/likely pathogenic variants in X-linked genes, which caused ~ 1.14% (22/1922) of cases in our deaf probands. Among 22 diagnosed patients, we identified all causative variants without recurring variants in the identified genes, 12 of which were novel. In addition, seven VUS were identified, and further functional assays are needed regarding these variants (Table 1).

As expected, most identified variants occurred in the *POU3F4* gene (~ 59%); the most common gene was related to DFNX2. Several smaller studies yielded similar results [21, 37, 38]. *PRPS1*, *SMPX*, *AIMF1*, and *COL4A6* were also detected, indicating that the etiological contributions of these genes to hereditary HL were extremely low. Among genes associated with syndromic forms, only 11 variants in 9 genes were identified. However, we suspect that their contributions were underestimated because HL is a single symptom that occurs in many syndromic diseases and can be masked by typical clinical spectra. Indeed, several limitations need to be noted when interpreting our results. First, some X-linked deafness genes were not included in the predefined panel containing 227 HL-related genes, such as *RPS6KA3*, *EFNB1*, *HDAC8*, *ARX*, and *ANOS1*, which may cause a negative molecular diagnosis for some patients. Second, many pathogenic variants affecting known deafness genes may go undetected using current diagnostic algorithms because they reside in non-coding (intronic and regulatory) sequences or unannotated exons. The percentages of the identified genetic causes, therefore, may be slightly under-represented accordingly. Overall, our study represented a preliminary evaluation of the aggregate contribution of X-linked genes to heritable HL (~ 1.14%). The findings also expanded the variant spectrum of known X-linked genes and illustrated the importance of NGS and TGS for identifying less common deafness-related genes.

### Genotype–phenotype relation

Mutations in X-linked NSHL-related genes generally exhibited representative audiological profiles or typical radiological changes, but the phenotypic features associated with specific mutations were diverse, ranging from isolated HL to severe syndromic diseases. The *PRPS1* gene encodes PRS-1, which catalyzes the synthesis of phosphoribosyl pyrophosphate (PRPP) from ATP and ribose-5-phosphate (R5P) [2]. PRS-1 is active as a hexamer, which consists of three homodimers arranged in a propeller-like shape, each with an active site (including binding sites for ATP and R5P) and two regulatory allosteric sites; these sites are involved in feedback inhibition of the enzyme [2, 18]. As described above (Fig. 3A), known alterations are dispersed throughout the *PRPS1* open reading frame and cause various disorders, indicating that each mutation results in a specific phenotype depending on its effects on the structure and function of PRS-1. Mutations destabilizing the ATP-binding site are associated with CMTX5, Arts syndrome, or intermediate syndrome [17, 39–41], whereas mutations affecting the allosteric sites abolish feedback regulation and contribute to PRS-1 superactivity (gain-of-function variants) [42, 43]. In DFNX1, mutations may exert their effects by disturbing interactions in the interface of the trimer or by damaging the local structure, but not destabilizing either the allosteric sites or the active site [2, 17]. Nevertheless, some authors have regarded PRS-1 loss-of-function as a unique condition associated with a phenotypic continuum, and the clinical presentation is highly dependent on the level of residual enzyme activity [44].

The *AIFM1* gene encodes mitochondrial apoptosis-inducing factor (AIF), a flavin adenine dinucleotide (FAD)-containing and nicotinamide adenine dinucleotide (NADH) -specific flavoprotein located in the mitochondrial intermembrane space [28]. *AIFM1* has at least two functions—a caspase-independent death effector and an FAD-dependent NADH oxidoreductase—with important roles in oxidative phosphorylation, redox control, respiratory chain activity, and the cell death pathway. Several recently identified mutations in human *AIFM1* lead to late-onset AN; there is no overlap with other *AIFM1* variations that cause syndromes (Fig. 3D). Based on phenotypic variability, it has been suggested that *AIFM1*-related diseases have distinct pathogenic mechanisms. In Cowchock syndrome, some mutations alter the redox properties of the mutant protein, resulting in increased apoptosis [45]. In mitochondrial encephalomyopathy, mutations can reduce the activity of respiratory chain complexes I–V and increase caspase-independent programmed cell death [46]. Conversely, in patients with distal motor neuropathy [47], some mutations do not enhance apoptosis and may be related to the defective mitochondrial respiration [29]. Concerning *AIFM1*-related AN, 17 identified mutations were mainly located in the NADH and second FAD domains, indicating the essential role of FAD-dependent NADH oxidoreductase in hearing function. Taken together, these results suggest that the phenotypic variability and severity of *AIFM1*-related disorders are dependent on the AIF feature predominantly affected (i.e., cellular level, structure, redox, or apoptogenic function) and the extent to which this feature is affected. However, further studies are required to clarify the pathogenesis of AN and the genotype–phenotype relationship.

Located on chromosome Xp22.12, the human *SMPX* gene encodes a small muscle protein with no known functional domains. The results of previous studies suggested that the presence of *SMPX* in hair cells plays a role in maintaining hair cell bundles [4, 5]. Mutations in *SMPX* led to DFNX4, and mainly caused truncated proteins, suggesting that a loss-of-function mechanism underlies this form of HL. Recently, four missense mutations were reported to result in distal myopathy with protein inclusions, and the pathological mechanism appears to be primarily based on aggregation of the mutant protein. Therefore, considering the genotypic and phenotypic features, the findings presented here suggest that the truncated variants cause X-linked NSHL through loss-of-function of mutant *SMPX*, in contrast to the gain-of-function mutations associated with distal myopathy [22]. Variations in *POU3F4* and *COL4A6* are reportedly associated with cochlear malformations. Concerning *POU3F4*, although approximately 100 variants have been identified, patients harboring *POU3F4* variants generally exhibit HL and typical IP-III. *COL4A6* has been extensively studied only in a Hungarian family, and the genotype–phenotype relationship requires further exploration [7].

### Diagnosis and treatment

Many human genetic syndromes include HL as a key feature. NSHL is present in 1–3 of every 1000 newborns with a monogenic form of non-syndromic, congenital, or early-onset HL; many more individuals develop postlingual HL [1, 36]. Unlike SHL, which can be classified because of its typical clinical spectrum, NSHL is extremely heterogeneous; it is often difficult to identify the disease-causing gene. Generally, most cases of autosomal recessive NSHL are prelingual HL involving severe to profound severity, whereas autosomal dominant NSHL is often observed in patients with postlingual progressive HL; such patients exhibit variation in age at onset and shape of audiograms [48]. However, many patients with X-linked NSHL have representative audiological profiles, typical cochlear malformations, or phenotypic differences across the sexes caused by skewed X chromosome inactivation. Therefore, predictions of the specific causative genes for X-linked NSHL based on phenotypic features are possible for these genes.

First, combined with HRCT, patients with radiological changes in the temporal bone have typical IP-III, and the phenotype is due to mutations in the *POU3F4* gene. The clinical features are often quite typical, facilitating diagnosis in most cases. Mutations in *COL4A6* should also be considered, although only the c.1771G>A variant has been reported to cause isolated malformed cochlea, with incomplete partition of the cochlea and incomplete separation from the IAC [7]. Second, in terms of audiological phenotypes, late-onset HL begins with effects on the low frequencies (up-slope audiogram) accompanied by anomalies or absence of ABR and the presence of DPOAE. *AIFM1* variations must be considered first because they constitute the most common genetic cause of all cases of noninfant-onset AN (~ 18.6%) [28]. Finally, HL caused by *PRPS1* and *SMPX* variants is progressive and severe, affecting all or high frequencies; its onset exhibits sex-related variation. It is difficult to tentatively predict the causative genes. However, based on X-linked inheritance in multiplex families, the disease-causing genes can be identified; *PRPS1* and *SMPX* variants can be further evaluated according to representative audiological profiles. Intriguingly, our NGS analysis did not identify any pathogenic mutations in known X-linked genes in a large X-linked family that included five male patients with deafness and cochlear malformation. We suspect that these symptoms were caused by mutations in novel X-linked deafness-related genes, and further studies are warranted.

Heritable HL affects all age groups from the newborn to the elderly, impairing language acquisition in children and causing social and vocational problems for adults. Patients with this condition need special attention and care in hearing rehabilitation. Current clinical treatments for hereditary HL are HA and CI. HA is often a first-line recommendation for patients with a milder degree of HL; CI may be a feasible option available for severe-to-profound HL when HA does not substantially improve hearing. However, the CI surgery and its effect varies due to the genetic basis, especially in DFNX genes. *POU3F4* mutation lead to typical IP-III, the malformed cochlea should be kept in mind during electrode choice. The Nucleus® Slim Straight Electrode is ideal to prevent the electrode from entering the IAC; an electrode with a “cork” type stopper or complete rings appears to be also proposed for these cases; intra-operative CT could be utilized to ensure correct electrode positioning [20, 49, 50]. Similarly, these factors need to be considered in patients with *COL4A6* mutations, and CI surgery can be successful [32]. For *AIFM1*-related AN, the affected lesion sites would be located on the postsynapses; auditory dyssynchrony progressively worsened over time, and some patients presented with CNH; these results suggested that CI may meet with limited success [6, 28]. Gene therapy may provide possibility for the treatment of *AIFM1*-related AN, but the approach does not be explored in X-linked deafness-related genes [51]. Overall, the treatment modalities for X-linked NSHL need to be individually tailored depending on the degree and type of HL and cause. The identification of genetic basis will be helpful for precision diagnosis and treatment decisions.

## Conclusion

Overall, 52.72% of the Chinese HL population showed positive molecular diagnoses; the aggregate contribution of X-linked genes was ~ 1.14%, and *POU3F4* variants caused ~ 59% of these diagnosed cases. The 15 novel variants reported here expanded the mutational spectrum of these genes. The discussion of genotype–phenotype relationships of DFNX genes provided a comprehensive clinical reference that will allow healthcare providers to make genotypic-based decisions when evaluating symptoms, thus providing better and more cost-effective patient care and preventive strategy in high-risk families.

## Supplementary Information

**Additional file 1: Table S1.** The 227 HL-related genes included in the predefined panel.

**Additional file 2: Table S2.** Overview of pathogenic variants and phenotypes in patients with variants in *PRPS1* gene.

**Additional file 3: Table S3.** Overview of pathogenic variants identified in *SMPX* gene.

**Additional file 4: Table S4.** Clinical phenotypes of the *AIFM1*-positive auditory neuropathy cases.

## Data Availability

All data generated or analysed during this study are included in the manuscript and its supplementary information files.

## Abbreviations

NSHL: Non-syndromic hearing loss
NGS: Next-generation sequencing
TGS: Third-generation sequencing
PTA: Pure tone audiometry
ABR: Auditory brainstem response
DPOAE: Distortion product otoacoustic emission
WES: Whole-exome sequencing
IP-III: Incomplete partition type III
AN: Auditory neuropathy
IAC: Internal auditory canal
CMTX5: Charcot-Marie-Tooth disease-5
PRS-1: Phosphoribosyl pyrophosphate synthetase 1
CI: Cochlear implants
HA: Hearing aids
CLS: Coffin–Lowry syndrome
ASI: Alport syndrome I
CD: Craniofrontonasal dysplasia
CDLS: Cornelia de Lange syndrome
FAD: Flavin adenine dinucleotide
NADH: Nicotinamide adenine dinucleotide

## References

[1] Smith RJ, Bale JF Jr, White KR. Sensorineural hearing loss in children. Lancet. 2005;365(9462):879–90. 15752533 10.1016/S0140-6736(05)71047-3

[2] Liu X, Han D, Li J, Han B, Ouyang X, Cheng J, Li X, Jin Z, Wang Y, Bitner-Glindzicz M, et al. Loss-of-function mutations in the PRPS1 gene cause a type of nonsyndromic X-linked sensorineural deafness, DFN2. Am J Hum Genet. 2010;86(1):65–71. 20021999 10.1016/j.ajhg.2009.11.015 PMC2801751

[3] de Kok YJ, van der Maarel SM, Bitner-Glindzicz M, Huber I, Monaco AP, Malcolm S, Pembrey ME, Ropers HH, Cremers FP. Association between X-linked mixed deafness and mutations in the POU domain gene POU3F4. Science. 1995;267(5198):685–8. 7839145 10.1126/science.7839145

[4] Schraders M, Haas SA, Weegerink NJ, Oostrik J, Hu H, Hoefsloot LH, Kannan S, Huygen PL, Pennings RJ, Admiraal RJ, et al. Next-generation sequencing identifies mutations of SMPX, which encodes the small muscle protein, X-linked, as a cause of progressive hearing impairment. Am J Hum Genet. 2011;88(5):628–34. 21549342 10.1016/j.ajhg.2011.04.012 PMC3146715

[5] Huebner AK, Gandia M, Frommolt P, Maak A, Wicklein EM, Thiele H, Altmüller J, Wagner F, Viñuela A, Aguirre LA, et al. Nonsense mutations in SMPX, encoding a protein responsive to physical force, result in X-chromosomal hearing loss. Am J Hum Genet. 2011;88(5):621–7. 21549336 10.1016/j.ajhg.2011.04.007 PMC3146719

[6] Zong L, Guan J, Ealy M, Zhang Q, Wang D, Wang H, Zhao Y, Shen Z, Campbell CA, Wang F, et al. Mutations in apoptosis-inducing factor cause X-linked recessive auditory neuropathy spectrum disorder. J Med Genet. 2015;52(8):523–31. 25986071 10.1136/jmedgenet-2014-102961 PMC4518735

[7] Rost S, Bach E, Neuner C, Nanda I, Dysek S, Bittner RE, Keller A, Bartsch O, Mlynski R, Haaf T, et al. Novel form of X-linked nonsyndromic hearing loss with cochlear malformation caused by a mutation in the type IV collagen gene COL4A6. Eur J Hum Genet. 2014;22(2):208–15. 23714752 10.1038/ejhg.2013.108 PMC3895628

[8] Corvino V, Apisa P, Malesci R, Laria C, Auletta G, Franzé A. X-linked sensorineural hearing loss: a literature review. Curr Genom. 2018;19(5):327–38. 10.2174/1389202919666171218163046 PMC6030855 30065609

[9] Sommen M, Schrauwen I, Vandeweyer G, Boeckx N, Corneveaux JJ, van den Ende J, Boudewyns A, De Leenheer E, Janssens S, Claes K, et al. DNA diagnostics of hereditary hearing loss: a targeted resequencing approach combined with a mutation classification system. Hum Mutat. 2016;37(8):812–9. 27068579 10.1002/humu.22999

[10] Li MM, Tayoun AA, DiStefano M, Pandya A, Rehm HL, Robin NH, Schaefer AM, Yoshinaga-Itano C. Clinical evaluation and etiologic diagnosis of hearing loss: a clinical practice resource of the American College of Medical Genetics and Genomics (ACMG). Genet Med. 2022;24(7):1392–406. 35802133 10.1016/j.gim.2022.03.018

[11] Yuan Y, Li Q, Su Y, Lin Q, Gao X, Liu H, Huang S, Kang D, Todd NW, Mattox D, et al. Comprehensive genetic testing of Chinese SNHL patients and variants interpretation using ACMG guidelines and ethnically matched normal controls. Eur J Hum Genet. 2020;28(2):231–43. 31541171 10.1038/s41431-019-0510-6 PMC6974605

[12] Wu J, Cao Z, Su Y, Wang Y, Cai R, Chen J, Gao B, Han M, Li X, Zhang D, et al. Molecular diagnose of a large hearing loss population from China by targeted genome sequencing. J Hum Genet. 2022;67(11):643–9. 35982127 10.1038/s10038-022-01066-5 PMC9592555

[13] Tarasov A, Vilella AJ, Cuppen E, Nijman IJ, Prins P. Sambamba: fast processing of NGS alignment formats. Bioinformatics. 2015;31(12):2032–4. 25697820 10.1093/bioinformatics/btv098 PMC4765878

[14] DePristo MA, Banks E, Poplin R, Garimella KV, Maguire JR, Hartl C, Philippakis AA, del Angel G, Rivas MA, Hanna M, et al. A framework for variation discovery and genotyping using next-generation DNA sequencing data. Nat Genet. 2011;43(5):491–8. 21478889 10.1038/ng.806 PMC3083463

[15] Oza AM, DiStefano MT, Hemphill SE, Cushman BJ, Grant AR, Siegert RK, Shen J, Chapin A, Boczek NJ, Schimmenti LA, et al. Expert specification of the ACMG/AMP variant interpretation guidelines for genetic hearing loss. Hum Mutat. 2018;39(11):1593–613. 30311386 10.1002/humu.23630 PMC6188673

[16] Mercati O, Abi Warde MT, Lina-Granade G, Rio M, Heide S, de Lonlay P, Ceballos-Picot I, Robert MP, Couloigner V, Beltrand J, et al. PRPS1 loss-of-function variants, from isolated hearing loss to severe congenital encephalopathy: new cases and literature review. Eur J Med Genet. 2020;63(11):104033. 32781272 10.1016/j.ejmg.2020.104033

[17] Robusto M, Fang M, Asselta R, Castorina P, Previtali SC, Caccia S, Benzoni E, De Cristofaro R, Yu C, Cesarani A, et al. The expanding spectrum of PRPS1-associated phenotypes: three novel mutations segregating with X-linked hearing loss and mild peripheral neuropathy. Eur J Hum Genet. 2015;23(6):766–73. 25182139 10.1038/ejhg.2014.168 PMC4270732

[18] Gandía M, Fernández-Toral J, Solanellas J, Domínguez-Ruiz M, Gómez-Rosas E, Del Castillo FJ, Villamar M, Moreno-Pelayo MA, Del Castillo I. Mutations in PRPS1 causing syndromic or nonsyndromic hearing impairment: intrafamilial phenotypic variation complicates genetic counseling. Pediatr Res. 2015;78(1):97–102. 25785835 10.1038/pr.2015.56

[19] Kim SY, Kim AR, Kim NK, Lee C, Han JH, Kim MY, Jeon EH, Park WY, Mittal R, Yan D, et al. Functional characterization of a novel loss-of-function mutation of PRPS1 related to early-onset progressive nonsyndromic hearing loss in Koreans (DFNX1): Potential implications on future therapeutic intervention. J Gene Med. 2016;18(11–12):353–8. 27886419 10.1002/jgm.2935 PMC5281059

[20] Su Y, Gao X, Huang SS, Mao JN, Huang BQ, Zhao JD, Kang DY, Zhang X, Dai P. Clinical and molecular characterization of POU3F4 mutations in multiple DFNX2 Chinese families. BMC Med Genet. 2018;19(1):157. 30176854 10.1186/s12881-018-0630-9 PMC6122742

[21] Bernardinelli E, Huber F, Roesch S, Dossena S. Clinical and molecular aspects associated with defects in the transcription factor POU3F4: a review. Biomedicines. 2023;11(6):1695. 37371790 10.3390/biomedicines11061695 PMC10296620

[22] Johari M, Sarparanta J, Vihola A, Jonson PH, Savarese M, Jokela M, Torella A, Piluso G, Said E, Vella N, et al. Missense mutations in small muscle protein X-linked (SMPX) cause distal myopathy with protein inclusions. Acta Neuropathol. 2021;142(2):375–93. 33974137 10.1007/s00401-021-02319-x PMC8270885

[23] Niu Z, Feng Y, Mei L, Sun J, Wang X, Wang J, Hu Z, Dong Y, Chen H, He C, et al. A novel frameshift mutation of SMPX causes a rare form of X-linked nonsyndromic hearing loss in a Chinese family. PLoS ONE. 2017;12(5):e0178384. 28542515 10.1371/journal.pone.0178384 PMC5444825

[24] Abdelfatah N, Merner N, Houston J, Benteau T, Griffin A, Doucette L, Stockley T, Lauzon JL, Young TL. A novel deletion in SMPX causes a rare form of X-linked progressive hearing loss in two families due to a founder effect. Hum Mutat. 2013;34(1):66–9. 22911656 10.1002/humu.22205

[25] Lv Y, Gu J, Qiu H, Li H, Zhang Z, Yin S, Mao Y, Kong L, Liang B, Jiang H, et al. Whole-exome sequencing identifies a donor splice-site variant in SMPX that causes rare X-linked congenital deafness. Mol Genet Genom Med. 2019;7(11):e967. 10.1002/mgg3.967 PMC6825843 31478598

[26] Deng Y, Niu Z, Fan L, Ling J, Chen H, Cai X, Mei L, He C, Zhang X, Wen J, et al. A novel mutation in the SMPX gene associated with X-linked nonsyndromic sensorineural hearing loss in a Chinese family. J Hum Genet. 2018;63(6):723–30. 29559740 10.1038/s10038-018-0443-x

[27] Gao S, Jiang Y, Wang G, Yuan Y, Huang S, Gao X, Li X, Zhang D, Wu J, Ji X, et al. Skewed X-chromosome inactivation and next-generation sequencing to identify a novel SMPX variants associated with X-linked hearing loss in a Chinese family. Int J Pediatr Otorhinolaryngol. 2018;113:88–93. 30174017 10.1016/j.ijporl.2018.07.022

[28] Wang H, Bing D, Li J, Xie L, Xiong F, Lan L, Wang D, Guan J, Wang Q. High frequency of AIFM1 variants and phenotype progression of auditory neuropathy in a Chinese population. Neural Plast. 2020;2020:5625768. 32684920 10.1155/2020/5625768 PMC7350177

[29] Wang R, Bai X, Yang H, Ma J, Yu S, Lu Z. Identification of a novel AIFM1 variant from a Chinese family with auditory neuropathy. Front Genet. 2022;13:1064823. 36479253 10.3389/fgene.2022.1064823 PMC9721464

[30] Kawarai T, Yamazaki H, Yamakami K, Tsukamoto-Miyashiro A, Kodama M, Rumore R, Caltagirone C, Nishino I, Orlacchio A. A novel AIFM1 missense mutation in a Japanese patient with ataxic sensory neuronopathy and hearing impairment. J Neurol Sci. 2020;409:116584. 31783324 10.1016/j.jns.2019.116584

[31] Bitner-Glindzicz M, Turnpenny P, Höglund P, Kääriäinen H, Sankila EM, van der Maarel SM, de Kok YJ, Ropers HH, Cremers FP, Pembrey M, et al. Further mutations in brain 4 (POU3F4) clarify the phenotype in the X-linked deafness, DFN3. Hum Mol Genet. 1995;4(8):1467–9. 7581392 10.1093/hmg/4.8.1467

[32] O’Brien A, Aw WY, Tee HY, Naegeli KM, Bademci G, Tekin M, Arnos K, Pandya A. Confirmation of COL4A6 variants in X-linked nonsyndromic hearing loss and its clinical implications. Eur J Hum Genet. 2022;30(1):7–12. 33840813 10.1038/s41431-021-00881-2 PMC8738723

[33] Petersen MB, Wang Q, Willems PJ. Sex-linked deafness. Clin Genet. 2008;73(1):14–23. 18005182 10.1111/j.1399-0004.2007.00913.x

[34] Reardon W. Sex linked deafness: wilde revisited. J Med Genet. 1990;27(6):376–9. 2359100 10.1136/jmg.27.6.376 PMC1017135

[35] Fraser GR. Sex-linked recessive congenital deafness and the excess of males in profound childhood deafness. Ann Hum Genet. 1965;29(2):171–96. 5865628 10.1111/j.1469-1809.1965.tb00512.x

[36] Del Castillo I, Morín M, Domínguez-Ruiz M, Moreno-Pelayo MA. Genetic etiology of non-syndromic hearing loss in Europe. Hum Genet. 2022;141(3–4):683–96. 35044523 10.1007/s00439-021-02425-6

[37] Huang BQ, Zeng JL, Yuan YY, Dai P. A novel mutation in POU3F4 in a Chinese family with X-linked non-syndromic hearing loss. J Otol. 2015;10(2):78–82. 29937786 10.1016/j.joto.2015.09.004 PMC6002573

[38] Bach I, Brunner HG, Beighton P, Ruvalcaba RH, Reardon W, Pembrey ME, van der Velde-Visser SD, Bruns GA, Cremers CW, Cremers FP, et al. Microdeletions in patients with gusher-associated, X-linked mixed deafness (DFN3). Am J Hum Genet. 1992;51(1):38–44. 1609803 PMC1682865

[39] Kim HJ, Sohn KM, Shy ME, Krajewski KM, Hwang M, Park JH, Jang SY, Won HH, Choi BO, Hong SH, et al. Mutations in PRPS1, which encodes the phosphoribosyl pyrophosphate synthetase enzyme critical for nucleotide biosynthesis, cause hereditary peripheral neuropathy with hearing loss and optic neuropathy (cmtx5). Am J Hum Genet. 2007;81(3):552–8. 17701900 10.1086/519529 PMC1950833

[40] de Brouwer AP, Williams KL, Duley JA, van Kuilenburg AB, Nabuurs SB, Egmont-Petersen M, Lugtenberg D, Zoetekouw L, Banning MJ, Roeffen M, et al. Arts syndrome is caused by loss-of-function mutations in PRPS1. Am J Hum Genet. 2007;81(3):507–18. 17701896 10.1086/520706 PMC1950830

[41] Synofzik M, Müller vom Hagen J, Haack TB, Wilhelm C, Lindig T, Beck-Wödl S, Nabuurs SB, van Kuilenburg AB, de Brouwer AP, Schöls L. X-linked Charcot-Marie-Tooth disease, Arts syndrome, and prelingual non-syndromic deafness form a disease continuum: evidence from a family with a novel PRPS1 mutation. Orphanet J Rare Dis. 2014;9:24. 24528855 10.1186/1750-1172-9-24 PMC3931488

[42] Chen P, Li J, Ma J, Teng M, Li X. A small disturbance, but a serious disease: the possible mechanism of D52H-mutant of human PRS1 that causes gout. IUBMB Life. 2013;65(6):518–25. 23509005 10.1002/iub.1154

[43] Becker MA, Smith PR, Taylor W, Mustafi R, Switzer RL. The genetic and functional basis of purine nucleotide feedback-resistant phosphoribosylpyrophosphate synthetase superactivity. J Clin Investig. 1995;96(5):2133–41. 7593598 10.1172/JCI118267 PMC185862

[44] de Brouwer AP, van Bokhoven H, Nabuurs SB, Arts WF, Christodoulou J, Duley J. PRPS1 mutations: four distinct syndromes and potential treatment. Am J Hum Genet. 2010;86(4):506–18. 20380929 10.1016/j.ajhg.2010.02.024 PMC2850427

[45] Rinaldi C, Grunseich C, Sevrioukova IF, Schindler A, Horkayne-Szakaly I, Lamperti C, Landouré G, Kennerson ML, Burnett BG, Bönnemann C, et al. Cowchock syndrome is associated with a mutation in apoptosis-inducing factor. Am J Hum Genet. 2012;91(6):1095–102. 23217327 10.1016/j.ajhg.2012.10.008 PMC3516602

[46] Ghezzi D, Sevrioukova I, Invernizzi F, Lamperti C, Mora M, D’Adamo P, Novara F, Zuffardi O, Uziel G, Zeviani M. Severe X-linked mitochondrial encephalomyopathy associated with a mutation in apoptosis-inducing factor. Am J Hum Genet. 2010;86(4):639–49. 20362274 10.1016/j.ajhg.2010.03.002 PMC2850437

[47] Tu H, Zhang A, Fu X, Xu S, Bai X, Wang H, Gao J. SMPX deficiency causes stereocilia degeneration and progressive hearing loss in CBA/CaJ mice. Front Cell Dev Biol. 2021;9:750023. 34722533 10.3389/fcell.2021.750023 PMC8551870

[48] Aldè M, Cantarella G, Zanetti D, Pignataro L, La Mantia I, Maiolino L, Ferlito S, Di Mauro P, Cocuzza S, Lechien JR, et al. Autosomal dominant non-syndromic hearing loss (DFNA): a comprehensive narrative review. Biomedicines. 2023;11(6):1616. 37371710 10.3390/biomedicines11061616 PMC10296186

[49] Sennaroğlu L, Bajin MD. Incomplete partition type III: A rare and difficult cochlear implant surgical indication. Auris Nasus Larynx. 2018;45(1):26–32. 28318810 10.1016/j.anl.2017.02.006

[50] Sennaroğlu L, Atay G, Bajin MD. A new cochlear implant electrode with a “cork”-type stopper for inner ear malformations. Auris Nasus Larynx. 2014;41(4):331–6. 24560093 10.1016/j.anl.2013.12.011

[51] Petit C, Bonnet C, Safieddine S. Deafness: from genetic architecture to gene therapy. Nat Rev Genet. 2023;24(10):665–86. 37173518 10.1038/s41576-023-00597-7

