# Supplementary material for: Genomic and phenotypic landscapes of X-linked hereditary hearing loss in the Chinese population
Source: Orphanet J Rare Dis. 2024 Sep 13;19:342. doi: 10.1186/s13023-024-03338-z (PMC11396341; doi:10.1186/s13023-024-03338-z)
Supplement: Supplementary file 1 — Additional file 1: Table S1. The 227 HL-related genes included in the predefined panel. [file 13023_2024_3338_MOESM1_ESM.docx]

Table S1. The 227 HL-related genes included in the predefined panel.

| Gene | Loci | Transcript | MIM | |
| --- | --- | --- | --- | --- |
| **Autosomal recessive non-syndromic HL(ARNSHL) genes (60)** | | | | |
| *GJB2* | DFNB1A/DFNA3A | NM_004004.6 | | 121011 |
| *GJB6* | DFNB1B/DFNA3B | NM_006783.4 | | 604418 |
| *MYO7A* | DFNB2/DFNA11/USH1B | NM_000260.4 | | 275903 |
| *MYO15A* | DFNB3 | NM_016239.4 | | 602666 |
| *SLC26A4* | DFNB4 | NM_000441.2 | | 605646 |
| *TMIE* | DFNB6 | NM_147196.2 | | 607237 |
| *TMC1* | DFNB7/11/DFNA36 | NM_138691.2 | | 606706 |
| *TMPRSS3* | DFNB8/10 | NM_024022.3 | | 605511 |
| *OTOF* | DFNB9 | NM_194248.3 | | 603681 |
| *CDH23* | DFNB12/USH1D | NM_022124.6 | | 605516 |
| *GIPC3* | DFNB15/72/95 | NM_133261.3 | | 608792 |
| *STRC* | DFNB16 | NM_153700.2 | | 606440 |
| *USH1C* | DFNB18/USH1C | NM_153676.4 | | 605242 |
| *OTOG* | DFNB18B | NM_001277269.1 | | 604487 |
| *TECTA* | DFNB21/DFNA8/12 | NM_005422.2 | | 602574 |
| *OTOA* | DFNB22 | NM_144672.4 | | 607038 |
| *PCDH15* | DFNB23/USH1F | NM_033056.4 | | 6055514 |
| *RDX* | DFNB24 | NM_002906.3 | | 179410 |
| *GRXCR1* | DFNB25 | NM_001080476.2 | | 613283 |
| *TRIOBP* | DFNB28 | NM_001039141.3 | | 609761 |
| *CLDN14* | DFNB29 | NM_144492.3 | | 605608 |
| *MYO3A* | DFNB30 | NM_017433.5 | | 606808 |
| *WHRN* | DFNB31/USH2D | NM_015404.4 | | 607928 |
| *CDC14A* | DFNB32/105 | NM_033312.2 | | 603504 |
| *ESRRB* | DFNB35 | NM_004452.3 | | 602167 |
| *ESPN* | DFNB36 | NM_031475.3 | | 606351 |
| *MYO6* | DFNB37/DFNA22 | NM_004999.4 | | 600970 |
| *HGF* | DFNB39 | NM_000601.6 | | 142409 |
| *ILDR1* | DFNB42 | NM_001199799.2 | | 609739 |
| *ADCY1* | DFNB44 | NM_021116.4 | | 103072 |
| *CIB2* | DFNB48 | NM_006383.4 | | 605564 |
| *MARVELD2* | DFNB49 | NM_001038603.3 | | 610572 |
| *COL11A2* | DFNB53/DFNA13/STL3 | NM_080680.2 | | 120290 |
| *PDZD7* | DFNB57 | NM_001195263.2 | | 612971 |
| *PJVK* | DFNB59 | NM_001042702.4 | | 610219 |
| *SLC26A5* | DFNB61 | NM_198999.3 | | 604943 |
| *LRTOMT* | DFNB63 | NM_001145308.4 | | 612414 |
| *DCDC2* | DFNB66 | NM_016356.5 | | 605755 |
| *LHFPL5* | DFNB66/67 | NM_182548.4 | | 609427 |
| *S1PR2* | DFNB68 | NM_004230.4 | | 605111 |
| *PNPT1* | DFNB70 | NM_033109.5 | | 610316 |
| *BSND* | DFNB73 | NM_057176.3 | | 606412 |
| *MSRB3* | DFNB74 | NM_001031679.3 | | 613719 |
| *SYNE4* | DFNB76 | NM_001039876.3 | | 615535 |
| *LOXHD1* | DFNB77 | NM_144612.6 | | 613072 |
| *TPRN* | DFNB79 | NM_001128228.3 | | 613354 |
| *GPSM2* | DFNB82 | NM_013296.5 | | 609245 |
| *PTPRQ* | DFNB84/DFNA73 | NM_001145026.2 | | 603317 |
| *OTOGL* | DFNB84 | NM_173591.3 | | 614925 |
| *TBC1D24* | DFNB86/DFNA65 | NM_001199107.2 | | 613577 |
| *ELMOD3* | DFNB88 | NM_001135022.2 | | 615427 |
| *KARS* | DFNB89 | NM_001130089.1 | | 601421 |
| *SERPINB6* | DFNB91 | NM_004568.5 | | 173321 |
| *CABP2* | DFNB93 | NM_016366.3 | | 607314 |
| *MET* | DFNB97 | NM_000245.4 | | 164860 |
| *TSPEAR* | DFNB98 | NM_144991.3 | | 612920 |
| *GRXCR2* | DFNB101 | NM_001080516.1 | | 615752 |
| *EPS8* | DFNB102 | NM_004447.6 | | 600206 |
| *CLIC5* | DFNB103 | NM_001114086.2 | | 607293 |
| *FAM65B* | DFNB104 | NM_014722.5 | | 611410 |
| **Autosomal dominant non-syndromic HL(ADNSHL) genes (27)** | | | | |
| *DIAPH1* | DFNA1 | NM_005219.5 | | 602121 |
| *KCNQ4* | DFNA2A | NM_004700.4 | | 603537 |
| *GJB3* | DFNA2B | NM_024009.3 | | 603324 |
| *IFNLR1* | DFNA2C | NM_173064.3 | | 607404 |
| *MYH14* | DFNA4A | NM_024729.3 | | 608568 |
| *CEACAM16* | DFNA4B | NM_001039213.4 | | 614591 |
| *GSDME* | DFNA5 | NM_004403.3 | | 608798 |
| *WFS1* | DFNA6/14/38 | NM_006005.3 | | 606201 |
| *COCH* | DFNA9 | NM_004086.3 | | 603196 |
| *EYA4* | DFNA10 | NM_004100.5 | | 603550 |
| *POU4F3* | DFNA15 | NM_002700.3 | | 602460 |
| *MYH9* | DFNA17 | NM_002473.5 | | 160775 |
| *ACTG1* | DFNA20/26 | NM_001614.5 | | 102560 |
| *SIX1* | DFNA23/BOR3 | NM_005982.4 | | 601205 |
| *SLC17A8* | DFNA25 | NM_139319.3 | | 607557 |
| *GRHL2* | DFNA28 | NM_024915.4 | | 608576 |
| *NLRP3* | DFNA34 | NM_004895.4 | | 606416 |
| *COL11A1* | DFNA37/STL2 | NM_001854.4 | | 120280 |
| *CRYM* | DFNA40 | NM_001888.5 | | 123740 |
| *P2RX2* | DFNA41 | NM_174873.3 | | 600844 |
| *CCDC50* | DFNA44 | NM_178335.3 | | 611051 |
| *TJP2* | DFNA51 | NM_004817.4 | | 607709 |
| *TNC* | DFNA56 | NM_002160.4 | | 187380 |
| *DIABLO* | DFNA64 | NM_019887.6 | | 605219 |
| *OSBPL2* | DFNA67 | NM_144498.3 | | 606731 |
| *HOMER2* | DFNA68 | NM_004839.4 | | 604799 |
| *KITLG* | DFNA69 | NM_000899.5 | | 184745 |
| **X-link HL genes (5)** | | | | |
| *PRPS1* | DFNX1 | NM_002764.3 | | 311850 |
| *POU3F4* | DFNX2 | NM_000307.5 | | 300039 |
| *SMPX* | DFNX4 | NM_014332.3 | | 300226 |
| *AIFM1* | DFNX5 | NM_004208.4 | | 300169 |
| *COL4A6* | DFNX6 | NM_001847.4 | | 303631 |
| **Syndromic HL genes (34)** | | | | |
| *PAX3* | WS1 | NM_181457.4 | | 606597 |
| *MITF* | WS2A | NM_000248.3 | | 156845 |
| *SNAI2* | WS2D | NM_003068.5 | | 602150 |
| *SOX10* | WS2E/WS4C | NM_006941.3 | | 602229 |
| *EDNRB* | WS4A | NM_000115.5 | | 131244 |
| *EDN3* | WS4B | NM_207034.3 | | 131242 |
| *USH1G* | USH1G | NM_173477.5 | | 602150 |
| *USH2A* | USH2A | NM_206933.3 | | 608400 |
| *ADGRV1* | USH2C | NM_032119.4 | | 602851 |
| *CLRN1* | USH3A | NM_174878.3 | | 606397 |
| *HARS* | USH3B | NM_002109.6 | | 142810 |
| *TCOF1* | TCOF1 | NM_000356.4 | | 606847 |
| *POLR1D* | TCOF2 | NM_015972.4 | | 613715 |
| *POLR1C* | TCOF3 | NM_203290.4 | | 610060 |
| *COL2A1* | STL1 | NM_001844.5 | | 120140 |
| *COL9A1* | STL4 | NM_001851.5 | | 120210 |
| *COL9A2* | STL5 | NM_001852.4 | | 120260 |
| *HSD17B4* | PRLTS1 | NM_000414.4 | | 601860 |
| *HARS2* | PRLTS2 | NM_012208.4 | | 600783 |
| *CLPP* | PRLTS3/DFNB81 | NM_006012.4 | | 601119 |
| *LARS2* | PRLTS4 | NM_015340.4 | | 604544 |
| *TWNK* | PRLTS5 | NM_021830.5 | | 606075 |
| *FOXI1* |  | NM_012188.5 | | 601093 |
| *KCNJ10* |  | NM_002241.5 | | 602208 |
| *NDP* | NDP1 | NM_000266.4 | | 300658 |
| *KCNQ1* | JLNS1 | NM_000218.2 | | 607542 |
| *KCNE1* | JLNS2 | NM_000219.6 | | 176261 |
| *SEMA3E* |  | NM_012431.3 | | 608188 |
| *CHD7* |  | NM_017780.4 | | 608892 |
| *EYA1* | BOR1 | NM_000503.6 | | 601653 |
| *SIX5* | BOR2 | NM_175875.5 | | 600963 |
| *COL4A3* |  | NM_000091.4 | | 120070 |
| *COL4A4* |  | NM_000092.4 | | 120131 |
| *COL4A5* |  | NM_000495.5 | | 303630 |
| **Other HL related genes (101)** | | | | |
| *ABCD1* |  | NM_000033.4 | | 300371 |
| *ABHD12* |  | NM_001042472.3 | | 613599 |
| *ALMS1* |  | NM_015120.4 | | 606844 |
| *ALX3* |  | NM_006492.3 | | 606014 |
| *ALX4* |  | NM_021926.4 | | 605420 |
| *ANKH* |  | NM_054027.6 | | 605145 |
| *ATP2B2* |  | NM_001001331.4 | | 108733 |
| *ATP6V1B1* |  | NM_001692.4 | | 192132 |
| *ATP6V1B2* |  | NM_001693.4 | | 606939 |
| *BCAP31* |  | NM_001139441.1 | | 300398 |
| *BCOR* |  | NM_017745.6 | | 300485 |
| *BCS1L* |  | NM_004328.5 | | 603647 |
| *C5ORF42* |  | NM_023073.3 | | 614571 |
| *CACNA1D* |  | NM_000720.4 | | 114206 |
| *CD151* |  | NM_004357.5 | | 602243 |
| *CHM* |  | NM_000390.4 | | 300390 |
| *CISD2* |  | NM_001008388.5 | | 611507 |
| *CLCN7* |  | NM_001287.6 | | 602727 |
| *CLCNKA* |  | NM_004070.4 | | 602024 |
| *CLCNKB* |  | NM_000085.4 | | 602023 |
| *COL1A1* |  | NM_000088.3 | | 120150 |
| *COLEC11* |  | NM_024027.4 | | 612502 |
| *COQ6* |  | NM_182476.3 | | 614647 |
| *DCAF17* |  | NM_025000.4 | | 612515 |
| *DHODH* |  | NM_001361.5 | | 126064 |
| *DIAPH3* |  | NM_001042517.2 | | 614567 |
| *DNAJC3* |  | NM_006260.5 | | 601184 |
| *DNMT1* |  | NM_001130823.3 | | 126375 |
| *DSPP* |  | NM_014208.3 | | 125485 |
| *ECM1* |  | NM_004425.4 | | 602201 |
| *ERCC3* |  | NM_000122.1 | | 133510 |
| *FGF10* |  | NM_004465.2 | | 602115 |
| *FGF3* |  | NM_005247.4 | | 164950 |
| *FGFR1* |  | NM_023110.2 | | 136350 |
| *FGFR2* |  | NM_000141.4 | | 176943 |
| *FGFR3* |  | NM_000142.4 | | 134934 |
| *FLNA* |  | NM_001456.3 | | 300017 |
| *FOXC1* |  | NM_001453.3 | | 601090 |
| *FRAS1* |  | NM_025074.7 | | 607830 |
| *FREM2* |  | NM_207361.6 | | 608945 |
| *GALE* |  | NM_000403.4 | | 606953 |
| *GATA3* |  | NM_001002295.2 | | 131320 |
| *GJA1* |  | NM_000165.5 | | 121014 |
| *GRIP1* |  | NM_021150.4 | | 604597 |
| *HMX1* |  | NM_018942.3 | | 142992 |
| *HOXA2* |  | NM_006735.4 | | 604685 |
| *IARS2* |  | NM_018060.4 | | 612801 |
| *IGF1* |  | NM_000618.5 | | 147440 |
| *LHX3* |  | NM_014564.5 | | 600577 |
| *LRP2* |  | NM_004525.3 | | 600073 |
| *LRP5* |  | NM_002335.4 | | 603506 |
| *MAF* |  | NM_005360.5 | | 177075 |
| *MASP1* |  | NM_139125.3 | | 600521 |
| *MED12* |  | NM_005120.3 | | 300188 |
| *MGP* |  | NM_000900.5 | | 154870 |
| *MYO1A* |  | NM_005379.4 | | 601478 |
| *NF1* |  | NM_001042492.2 | | 613113 |
| *NF2* |  | NM_000268.3 | | 607379 |
| *NOTCH2* |  | NM_024408.4 | | 600275 |
| *OSTM1* |  | NM_014028.4 | | 607649 |
| *PDSS1* |  | NM_014317.5 | | 607429 |
| *PEX1* |  | NM_000466.3 | | 602136 |
| *PEX7* |  | NM_000288.4 | | 601757 |
| *PHYH* |  | NM_006214.4 | | 602026 |
| *PITX2* |  | NM_153427.2 | | 601542 |
| *PLEKHM1* |  | NM_014798.3 | | 611466 |
| *PLOD1* |  | NM_000302.4 | | 153454 |
| *PLOD3* |  | NM_001084.5 | | 603066 |
| *PMP22* |  | NM_000304.4 | | 601097 |
| *POLD1* |  | NM_002691.4 | | 174761 |
| *PRRX1* |  | NM_022716.4 | | 167420 |
| *PTPN11* |  | NM_002834.4 | | 176876 |
| *RECQL4* |  | NM_004260.3 | | 603780 |
| *RMND1* |  | NM_017909.4 | | 614917 |
| *SALL1* |  | NM_002968.2 | | 602218 |
| *SALL4* |  | NM_020436.5 | | 607343 |
| *SERAC1* |  | NM_032861.4 | | 614725 |
| *SF3B4* |  | NM_005850.5 | | 605593 |
| *SLC33A1* |  | NM_004733.4 | | 603690 |
| *SLC4A11* |  | NM_032034.3 | | 610206 |
| *SLITRK6* |  | NM_032229.3 | | 609681 |
| *SNX10* |  | NM_001199835.1 | | 614780 |
| *SOST* |  | NM_025237.3 | | 605740 |
| *SOX9* |  | NM_000346.4 | | 608160 |
| *SQSTM1* |  | NM_003900.5 | | 601530 |
| *TBX1* |  | NM_080647.1 | | 602054 |
| *TBX22* |  | NM_001109878.2 | | 300307 |
| *TCIRG1* |  | NM_006019.4 | | 604592 |
| *TCTN3* |  | NM_015631.6 | | 613847 |
| *TFAP2A* |  | NM_001032280.3 | | 107580 |
| *TGFB1* |  | NM_000660.7 | | 190180 |
| *TIMM8A* |  | NM_004085.4 | | 300356 |
| *TNFRSF11A* |  | NM_003839.4 | | 603499 |
| *TNFRSF11B* |  | NM_002546.4 | | 602643 |
| *TNFSF11* |  | NM_003701.4 | | 602642 |
| *TP63* |  | NM_003722.5 | | 603273 |
| *TRMU* |  | NM_018006.5 | | 610230 |
| *TSHZ1* |  | NM_005786.6 | | 614427 |
| *TWIST1* |  | NM_000474.4 | | 601622 |
| *TYR* |  | NM_000372.5 | | 606933 |
| *ZNF687* |  | NM_020832.3 | | 610568 |

Genes that cause both AR non-syndromic HL, AD non-syndromic HL and syndromic HL are listed under ARNSHL including: *GJB2, GJB6, MYO7A, TMC1, CDH23, USH1C, TECTA, PCDH15, MYO3A, WHRN, MYO6, COL11A2, PTPRQ, TBC1D24*; Genes that cause both ADNHSL and syndromic HL are listed under ADNSHL including: *SIX1, COL11A1*.
