# Supplementary material for: Genomic and phenotypic landscapes of X-linked hereditary hearing loss in the Chinese population
Source: Orphanet J Rare Dis. 2024 Sep 13;19:342. doi: 10.1186/s13023-024-03338-z (PMC11396341; doi:10.1186/s13023-024-03338-z)
Supplement: Supplementary file 2 — Additional file 2: Table S2. Overview of pathogenic variants and phenotypes in patients with variants in PRPS1 gene. [file 13023_2024_3338_MOESM2_ESM.docx]

| Nucleotide | Amino Acid | Location | Male | | Female | |
| --- | --- | --- | --- | --- | --- | --- |
|  |  |  | **Onset(y)** | **Phenotype of HL** | **Onset(y)** | **Phenotype of HL** |
| c.136G>C | P.E46Q | Exon2 | 8 | Progressive, bilateral, severe-profound | - | Normal |
| c.193G>A | p.D65N | Exon2 | 5-14 | Symmetric, progressive, sensorineural, severe to profound, all frequency | 40-55 | Asymmetric, symmetric or unilateral, Sensorineural, mild to moderate or severe, flat |
| c. 244G>C | p.A82P | Exon2 | 3-7 | Symmetric, progressive, sensorineural, moderate to profound, all frequency | - | Normal |
| c.259G>A | p.A87T | Exon2 | Congenital | Symmetric, profound, sensorineural, all frequency | NA | Mild to moderate,  especially in the high frequencies. |
| c.337G>T | p.A113S | Exon3 | Postlingual | Symmetric, bilateral, sensorineural, profound | Postlingual | Moderate |
| c.826C>T | p.P276S | Exon6 | NA | Symmetric, bilateral, sensorineural | NA | NA |
| c.824T>C | p.I275T | Exon6 | <14 | Symmetric, bilateral, sensorineural, moderate | - | Normal |
| c.869T>C | p.I290T | Exon7 | Congenital | Progressive, sensorineural, profound, all frequency | NA | Mild to moderate |
| c.916G>A | p.G306R | Exon7 | 7-20 | Symmetric, progressive, sensorineural, severe, low- and middle- frequency | NA | Mild to moderate |
| c.917G>A | p.G306E | Exon7 | Prelingual, | Bilateral, severe to profound, all frequency | - | Normal |

Table S2. Overview of pathogenic variants and phenotypes in patients with variants in *PRPS1* gene.

The references were listed in the manuscript [2, 16-19]. y: years old; NA, not available.
