# Supplementary material for: Genomic and phenotypic landscapes of X-linked hereditary hearing loss in the Chinese population
Source: Orphanet J Rare Dis. 2024 Sep 13;19:342. doi: 10.1186/s13023-024-03338-z (PMC11396341; doi:10.1186/s13023-024-03338-z)
Supplement: Supplementary file 3 — Additional file 3: Table S3. Overview of pathogenic variants identified in SMPX gene. [file 13023_2024_3338_MOESM3_ESM.docx]

Table S3. Overview of pathogenic variants identified in *SMPX* gene.

| Nucleotide | Amino Acid | Location | Male | | | Female | | |
| --- | --- | --- | --- | --- | --- | --- | --- | --- |
|  |  |  | **Onset(y)** | **Severity** | **Progressive** | **Onset(y)** | **Severity** | **Progressive** |
| c.29insA | p.N10Kfs*3 | Exon2 | 5–10 | Bilateral; profound | Yes | 20-30 | Bilateral; moderate to profound; | Yes |
| c.87dup | p.G30Rfs*12 | Exon3 | 7 | Bilateral; severe | Yes | >30 | Unilateral or bilateral; severe | Yes |
| c.99del | p.R34Efs*47 | Exon3 | <10 | Bilateral; severe | Yes | 4-62 | Unilateral or bilateral; variable severity | Yes |
| c.109G>T | p.E37* | Exon3 | 3–7 | Bilateral; moderate to profound | Yes | 11-30 | Bilateral; moderate to severe | Yes |
| c.130del | p.E44Rfs*37 | Exon3 | 4 | Bilateral; severe | Yes | - | - | - |
| c.132+1G>A | - | Intron3 | Newborn | Bilateral; severe to profound | Yes | >50 | Bilateral; moderate to severe | NA |
| c.132+1G>T | - | Intron3 | <10 | Bilateral; severe | Yes | - | - | - |
| c.133-1G>A | - | Intron3 | <18 | Bilateral; profound | Yes | <18 | Unilateral; severe | Yes |
| c.175G>T | p.G59* | Exon4 | 5–7 | Bilateral; moderate to profound | Yes | 31-40 | Bilateral; moderate | Yes |
| c.214G>T | p.E72* | Exon4 | 2–10 | Bilateral; moderate to profound | Yes | 3-48 | Unilateral or bilateral; mild to profound | Yes |
| c.217dup | p.I73Nfs*5 | Exon4 | 0–8 | Bilateral; severe to profound | Yes | 31-40 | Unilateral or bilateral; mild | - |
| c.262C>G | p.Q88E | Exon4 | <20 | Bilateral; mild to moderately severe | - | 3; 31-40 | Bilateral; mild to profound | Yes |

The references were listed in the manuscript [4, 5, 22-27]. y: years old; NA, not available.
