# Supplementary material for: Genomic and phenotypic landscapes of X-linked hereditary hearing loss in the Chinese population
Source: Orphanet J Rare Dis. 2024 Sep 13;19:342. doi: 10.1186/s13023-024-03338-z (PMC11396341; doi:10.1186/s13023-024-03338-z)
Supplement: Supplementary file 4 — Additional file 4: Table S4. Clinical phenotypes of the AIFM1-positive auditory neuropathy cases. [file 13023_2024_3338_MOESM4_ESM.docx]

Table S4. Clinical phenotypes of the *AIFM1*-positive auditory neuropathy cases.

| Patient ID | Sex | Onset (y) | Tested age (y) | Severity | Type of HL | AN | Other |
| --- | --- | --- | --- | --- | --- | --- | --- |
| 1 | M | 10 | 14 | Moderate | up-slope | Yes | Tinnitus, Myopia |
| 2 | M | 19 | 20 | Mild | up-slope | Yes | Unsteadiness, CNH, Numbness of extremities |
| 3 | M | 16 | 20 | Moderate | up-slope | Yes | Myopia |
| 4 | M | 14 | 21 | Moderate | other | Yes | CNH |
| 5 | M | 20 | 28 | Moderate | up-slope | Yes | Tinnitus, Unsteadiness, Numbness of extremities |
| 6 | M | 12 | 21 | Mild | up-slope | Yes | Tinnitus |
| 7 | M | 12 | 27 | Moderate | up-slope | Yes | Tinnitus, Unsteadiness, Numbness of extremities, Vertigo |
| 8 | M | 10 | 20 | Moderate | up-slope | Yes | Tinnitus, Vertigo |
| 9 | M | 15 | 28 | Moderate | up-slope | Yes | Tinnitus |
| 10 | M | 10 | 24 | Moderate | up-slope | Yes | - |
| 11 | M | 8 | 20 | Normal | up-slope /flat | Yes | - |
| 12 | M | 17 | 34 | Moderate | up-slope | Yes | Tinnitus |
| 13 | M | 14 | 17 | Moderate | up-slope | Yes | Tinnitus, Unsteadiness, Numbness of extremities, Myopia |
| 14 | M | 20 | 25 | Normal | up-slope /flat | Yes | Tinnitus |
| 15 | M | 14 | 18 | Mild | up-slope | Yes | Myopia |
| 16 | M | 14 | 21 | Mild | up-slope | Yes | Tinnitus, Vertigo, Myopia |
| 17 | M | 11 | 16 | Mild | up-slope | Yes | Tinnitus, Myopia, CNH |
| 18 | M | 18 | 31 | Mild | up-slope | Yes | Tinnitus, Unsteadiness, CNH, Numbness of extremities |
| 19 | M | 13 | 16 | Moderate | U-shape | Yes | Tinnitus, Unsteadiness, Numbness of extremities |
| 20 | M | 15 | 20 | Mild | up-slope | Yes | - |
| 21 | M | 14 | 36 | Moderate | up-slope | Yes | Tinnitus, Unsteadiness, CNH, Numbness of extremities, Myopia |
| 22 | M | 7 | 15 | Mild | U-shape | Yes | - |
| 23 | M | 16 | 27 | Moderate | flat | Yes | Tinnitus, Unsteadiness, CNH, Numbness of extremities |
| 24 | M | 6 | 21 | Mild | up-slope | Yes | Tinnitus |
| 25 | M | 8 | 20 | Mild | up-slope | Yes | Tinnitus, Myopia |
| 26 | M | 14 | 24 | Moderate | up-slope | Yes | - |
| 27 | M | 5 | 14 | Moderate | up-slope | Yes | Tinnitus |
| 28 | M | 11 | 15 | Mild | U-shape | Yes | Tinnitus, Myopia, CNH |
| 29 | M | 16 | 27 | Mild | up-slope | Yes | Unsteadiness, CNH, Numbness of extremities |
| 30 | M | 17 | 20 | Mild | up-slope | Yes | Tinnitus |
| 31 | M | 19 | 19 | Mild | up-slope | Yes | Tinnitus, Numbness of extremities |
| 32 | M | 13 | 15 | Moderate | up-slope | Yes | Tinnitus, CNH |
| 33 | F | 15 | 17 | Mild | up-slope | Yes | Tinnitus, Myopia |
| 34 | F | 18 | 21 | Moderate | up-slope | Yes | Tinnitus |
| 35 | F | 13 | 17 | Mild | flat/up-slope | Yes | - |
| 36 | F | 6 | 6 | Normal | flat/down-sloping | Yes | Tinnitus, Unsteadiness, Numbness of extremities |
| 37 | F | 21 | 28 | Mild | up-slope | Yes | Tinnitus, Myopia, CNH |

AN: Auditory neuropathy, CNH: cochlear nerve hypoplasia, y: years old, M: male, F: female. The references were listed in the manuscript [7, 28-30].
